# Rehabilitation at Home With the Development of a Sustainable Model Placing the Person’s Needs and Environment at Heart: Protocol for a Multimethod Project

**DOI:** 10.2196/56996

**Published:** 2024-07-23

**Authors:** Marie Elf, Lizette Norin, Louise Meijering, Hélène Pessah-Rasmussen, Riitta Suhonen, Magnus Zingmark, Maya Kylén

**Affiliations:** 1 School of Health and Welfare Dalarna University Falun Sweden; 2 Population Research Centre Faculty of Spatial Sciences University of Groningen Groningen Netherlands; 3 Department of Neurology, Rehabilitation Medicine, Memory disorders and Geriatrics Skåne University Hospital Malmö Sweden; 4 Department of Clinical Sciences Lund Lund University Lund Sweden; 5 Department of Nursing Science University of Turku Turku Finland; 6 Turku University Hospital The Wellbeing Services County of Southwest Finland Turku Finland; 7 Department of Community Medicine and Rehabilitation Umea University Umea Sweden; 8 Department of Health Sciences Lund University Lund Sweden

**Keywords:** co-design, early supported discharge, home, integrated care, life space mobility, multi-methods, physical environment, person-centered care, social environment, stroke rehabilitation

## Abstract

**Background:**

Each year, more than 1.5 million people in Europe have a stroke, and many experience disabilities leading to activity and participation restrictions. Home-based rehabilitation is the recommended approach for stroke rehabilitation, in line with the international shift to integrated care. Despite this, rehabilitation often focuses on the person’s physical functions, not the whole life situation and opportunities to live an active life. Given that rehabilitation today is often provided in the person’s home, there is a need to develop new models that consider the rehabilitation process as situated in the everyday living environment of persons with stroke. This project is grounded in experiences from our ongoing research, where we study the importance of the home environment for health and participation among persons with stroke, rehabilitated at home. This research has shown unmet needs, which lead to suboptimal rehabilitation outcomes. There is a need for studies on how to use environmental resources to optimize stroke rehabilitation in the home setting.

**Objective:**

The overarching objective of the project is to develop a new practice model for rehabilitation where the needs of the person are the starting point and where the environment is considered.

**Methods:**

The project will be conducted in partnership with persons with stroke, significant others, health care professionals, and care managers. Results from a literature review will form the base for interviews with the stakeholders, followed by co-designing workshops aiming to create a new practice model. Focus groups will be held to refine the outcome of the workshops to a practice model.

**Results:**

This 4-year project commenced in January 2023 and will continue until December 2026. The results of the literature review are, as of April 2024, currently being analyzed. The ethics application for the interviews and co-design phase was approved in October 2023 and data collection is ongoing during spring 2024. We aim to develop a practice model with stakeholders and refine it together with care managers and decision makers. The outcome is a new practice model and implementation plan, which will be achieved in autumn 2026.

**Conclusions:**

The project contributes with a prominent missing puzzle to optimize the rehabilitation process by adding a strong focus on user engagement combined with integrating different aspects of the environment. The goal is to improve quality of life and increase reintegration in society for the large group of people living with the aftermath of a stroke. By co-designing with multiple stakeholders, we expect the model to be feasible and sustainable. The knowledge from the project will also contribute to an increased awareness of the importance of the physical environment for sustainable health care. The findings will lay the foundation for future upscaling initiatives.

**International Registered Report Identifier (IRRID):**

DERR1-10.2196/56996

## Introduction

### Background

This project (InHome) focuses on developing an implementable practice model that uses person-centered care principles. Our primary goal is to improve the quality of life of people with stroke by integrating their environment into the rehabilitation process. The intervention is complex and requires careful development, evaluation, and formulation of practical implementation strategies before scaling up. In InHome, the concept of the physical environment is fundamental. It consists of the built environment (eg, stairs or doors), natural environment (outdoor surfaces), and social factors such as human interactions [1]. The environmental impact on a person’s health and ability to perform daily activities depends on the person’s cognitive and physical ability and the demands of the environment. The same environment can facilitate one activity for one person and be an obstacle for another.

### Life After Stroke

Each year, more than 1.5 million Europeans will have a stroke [2]. The number is expected to rise partly due to an aging population and improved survival rates [3,4]. Many stroke survivors face lasting cognitive, physical, emotional, and psychological challenges. This can be manifested as memory loss, communication difficulties, reduced mobility, and emotional impact such as depression and anxiety [5-7]. Such impairments can lead to decreased social interactions and limited community mobility, resulting in diminished participation in meaningful activities (eg, activities outside the home and leisure activities) and social engagements. This constrained living space not only impacts daily living and well-being but also poses a risk of isolating persons with stroke from essential social resources and services [8,9]. Moreover, studies have shown that people who have had a stroke tend to make fewer social trips compared with healthy adults in general [9,10]. These limitations have profound implications for daily life, health, and overall well-being, highlighting the critical importance of community integration in the rehabilitation of stroke survivors [11].

The overall complexity of life after stroke is often underestimated and requires a holistic and integrated support system [11,12]. This research underscores the necessity of including factors of the physical environment at home and in the neighborhood in rehabilitation, recognizing the profound influence of the environment on recovery and everyday life [11,13,14]. Issues, such as accessibility challenges and fall risks, are common [5], with an inaccessible living environment correlating with poorer health perceptions and recovery outcomes [13]. Addressing these environmental challenges is crucial, but equally important is the recognition of the individual’s role and agency in the recovery process. This dual focus on environmental adaptability and personal empowerment is essential for a comprehensive approach to poststroke care.

The recovery process poststroke extends beyond the hospital, and many feel abandoned and excluded from decision-making and rehabilitation planning, demonstrating a strong need for more person-centered approaches [5,15,16]. Research shows that recovery after a stroke is best supported when the person and family feel that they can manage daily life and control the rehabilitation and recovery process and activities with the support of resources in the person’s environment [17,18].

The environment plays a crucial role in supporting individuals who have experienced a stroke. An environment that allows individuals to control their choices and activities fosters a sense of autonomy, which is closely related to self-efficacy. Self-efficacy, an individual’s belief in their capabilities to execute behaviors necessary to make specific performance achievements [19], can significantly impact their motivation, well-being, and recovery outcomes. In that sense, an accessible environment reduces physical barriers, enabling individuals to perform daily tasks more independently. This sense of independence can enhance self-efficacy as the person recognizes their ability to manage daily life. Moreover, an environment that can be easily modified to suit the individual’s changing needs supports ongoing engagement in activities, promoting a sense of control and competence. A safe and secure environment reduces the fear of injury or fall, allowing the individual to engage in activities confidently, thus fostering a sense of self-efficacy. An environment that facilitates social interactions and support networks can provide encouragement, feedback, and assistance, all of which can bolster an individual’s self-efficacy.

### Stroke Care and Rehabilitation

In Sweden, a person with a stroke will meet a decentralized, fragmented, and complex health care system, with specialized rehabilitation managed by regional health care providers. After the acute phase, the responsibility for long-term support is shifting to the municipalities [20]. To mitigate these challenges and enhance integrated care, early supported discharge (ESD), an internationally recommended approach in stroke rehabilitation, is practiced in certain regions in Sweden. ESD involves persons with mild to moderate stroke continuing their rehabilitation at home guided by a multidisciplinary team [17].

Concurrently, there is a transformative shift in stroke care, with shorter hospital stays. This shift necessitates a broader responsibility for individuals and their families in managing poststroke recovery at home [21]. This transformation emphasizes the importance of patient empowerment, self-management of health, and collaborative decision-making with health care providers. Ensuring stroke survivors and their families are well-equipped to navigate these complexities is essential for optimizing recovery and quality of life [22,23]. Moreover, it highlights the crucial role of the home environment, which can simultaneously serve as a support and present a unique challenge [16,24]. For example, being at home in a familiar place can support the recovery process. It creates opportunities for rehabilitation in an environment meaningful to the person rather than the hospital, allowing problem-solving and planning in a meaningful context. However, people with stroke report that health service providers rarely ask about their home environment, and its impact on their ability to reintegrate into society after stroke is often overlooked during the rehabilitation process [16]. Instead, existing rehabilitation models focus on functional abilities rather than participation in meaningful activities. Further, the role played by the environment is regarded as a side finding in stroke research [25].

To sum up, after a stroke, individuals may face challenges spanning cognitive, physical, emotional, and psychological domains, often leading to reduced social interactions, limited mobility in society, and a subsequent reduction in participation in meaningful activities. The trend toward shorter hospital stays has shifted responsibility for poststroke care to the home environment, requiring robust support networks and resources. However, existing care models cannot meet these needs, especially when it comes to integrating the physical environment to support the person recovering from a stroke at home.

Thus, there is a critical gap in the rehabilitation process that addresses the complex and multifaceted needs of stroke survivors in their physical environment. This gap highlights the need for innovative and holistic models of care that are co-designed with input from people with stroke, relatives, and health care professionals. Such models must integrate the physical environment of the stroke survivor, ensuring tailored and practical support. To ensure feasibility and scalability, the Medical Research Council (MRC) recommends a step-by-step approach to the development, evaluation, and implementation of interventions. This approach ensures that interventions are robust, research-based, and ready for large-scale implementation [26]. In this new era of person-centered care, our initiative aims to bridge this gap, developing a holistic, co-designed practice model for poststroke rehabilitation in which the physical environment is integrated, and preparing it for widespread use and implementation.

### Study Objectives and Research Questions

InHome aims to develop a new practice model that integrates environmental factors in rehabilitation after a stroke for a more person-centered service. Following the MRC’s [23] recommendations for developing complex interventions, we will involve key stakeholders to develop and evaluate a new intervention aiming to improve health-related outcomes and support new effective working methods for rehabilitation services in the home.

The specific research questions are as follows: How do physical (ie, built, natural, and social) environmental factors contribute to supporting a person-centered rehabilitation process in the individual’s activity space? What methods and activities for integrating environmental factors to support a person-centered rehabilitation process are described in the literature? What experiences and expectations do persons with stroke, significant others, health care professionals, and care managers have of integrating environmental factors into the rehabilitation process? (2) How could a practice model that integrates physical environmental factors be designed to meet the expectations and requirements of persons with stroke and health care services, to make efficient use of resources for rehabilitation in the home? What environmental factors should be integrated into a new practice model to support a person-centered care rehabilitation process? How should a feasible practice model be designed? (3) What are the critical mechanisms for integrating environmental factors into stroke rehabilitation at home? How is the model perceived and evaluated by decision makers regarding its sustainability, for example, potential benefits including health outcomes, cost-effectiveness, resources, feasibility, and acceptability? What are the most important facilitators and obstacles to implementing the new practice model?

### Theoretical Framework

Several theoretical models focusing on person-environment interactions underpin InHome. The person-environment-occupation model [27], the Ecological Theory of Aging [28], and the International Classification of Functioning, Disability, and Health (ICF) [29] show that the environment comprises a multitude of facilitating or hindering factors external to the person. The models describe that a good fit between a person’s (P) functional abilities and the demands of environmental factors (E) leads to positive outcomes such as increased independence and overall well-being (P-E fit). Hence, to optimize rehabilitation outcomes, it is important to make use of a person’s environment and be aware of facilitating and hindering factors.

InHome is also grounded in Bandura’s social cognition theory and the key constructs of self-efficacy and collective self-efficacy [19]. These concepts see individuals as capable with unique experiences and resources and describe people’s or group’s beliefs about their ability to achieve specific performance levels, which then affects their control over life events [30].

**Figure 1:**
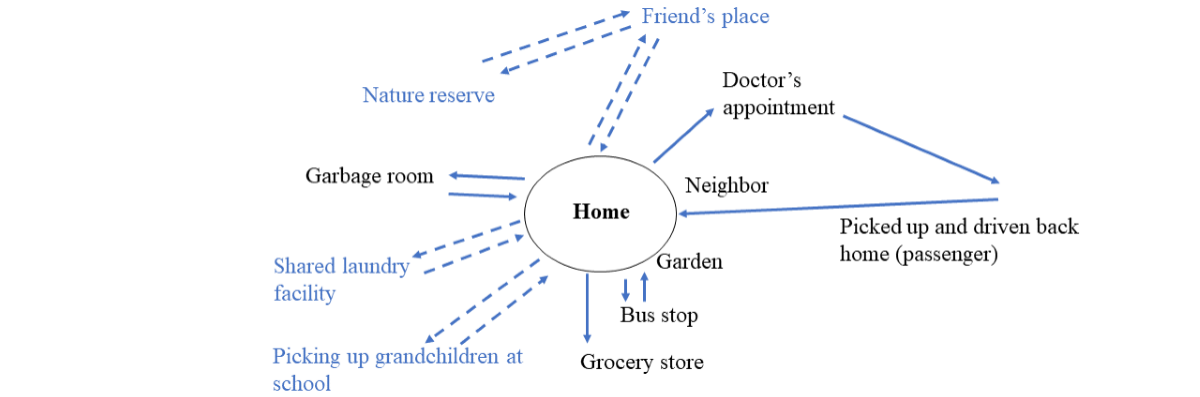
An example of how a person’s activity space might decrease in case of an illness like a stroke. Dotted blue lines represent meaningful places that are no longer accessible due to functional limitations and barriers related to the mode of transportation or the physical environment. Nondotted lines represent places that are still accessible.

Furthermore, we will use the concept of activity space, which has been defined as “the subset of all locations within which an individual has direct contact as a result of his or her day-to-day activities” [31] to operationalize the term physical environment. The value of the idea of activity space for our project lies in the fact that it is person-centered: it encompasses the area and locations that an individual uses, for different activities, such as work and leisure, using different modes of transport such as the car or bicycle. An individual’s activity space is likely to consist of different important nodes or locations from which activities are undertaken [32]. Typically, the most important node is the home, especially in populations who experience impairments in everyday life such as older adults [33]. The same is likely to apply to people affected by stroke. Figure 1 provides an abstract representation of an activity space.

## Methods

### Overview

**Figure 2:**
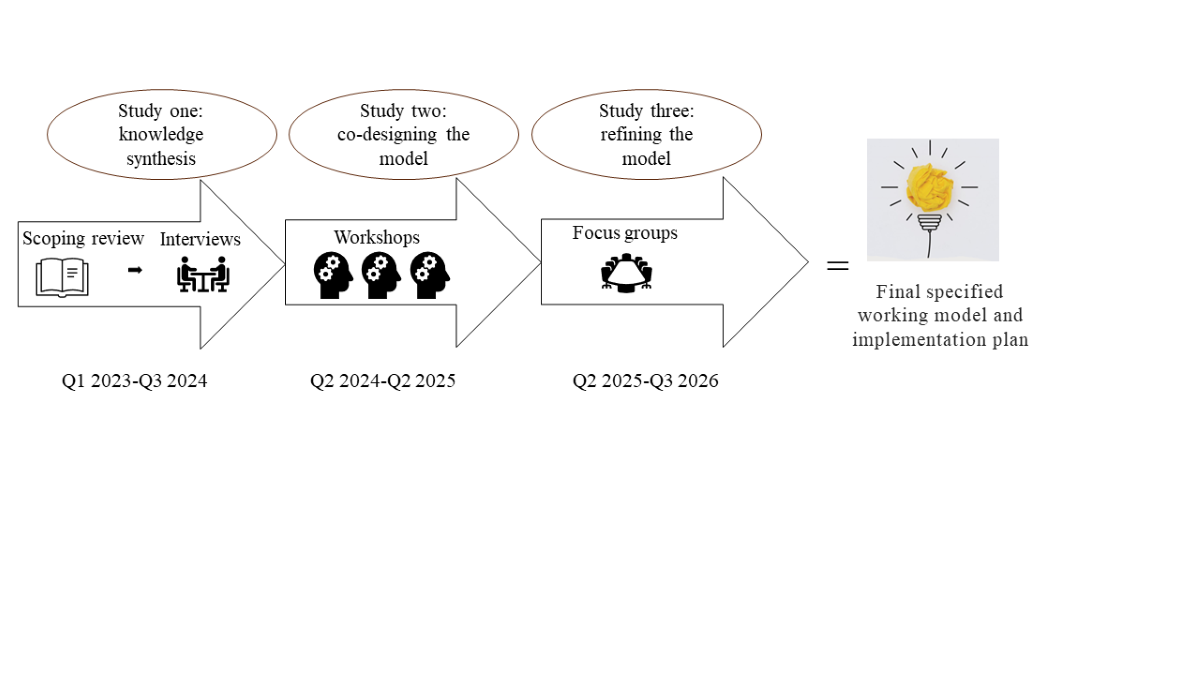
Illustration of the 3 studies in the multi-method project, InHome, aiming to develop a new working model for person-centered, home-based stroke rehabilitation integrating environmental factors.

InHome has a multimethod approach across 3 studies (Figure 2). We will use a research design that combines knowledge synthesis, and qualitative and co-design methods to investigate important environmental factors and implementation strategies for a new practice model that integrates environmental factors in the rehabilitation of people with stroke. To ensure a successful co-design process, we will use the PRODUCES (Problem Objective Design (end-) Users Co-creators Evaluation Scalability) [34] framework, which provides a systematic planning method for conducting, evaluating, and reporting research findings with specific stakeholder involvement and scientific consistency.

The first study, a knowledge synthesis, involves a scoping review which along with interviews aims to identify the critical factors for integrating environmental factors to support the person-centered rehabilitation process in the home. The knowledge synthesis will inform the following co-design of the intervention and implementation strategies, which is the second study. In the third study, we will involve the perspectives of decision makers to refine the model further. Figure 2 provides an overview of the project.

### Addressing Research Question 1: Knowledge Synthesis

#### Scoping Review

A total of 2 sources of data will be used to learn about essential environmental factors in the rehabilitation process and how those could be integrated into the process. First, we will conduct a scoping review [35] to update the evidence on the key environmental factors that are important to support a person-centered rehabilitation process in the home. The completeness of the review will be constrained by the availability of relevant published research, policy, and gray literature within the last 10 years (2012-2023). The search strategy will focus on the sensitivity of terms relating to rehabilitation at home (eg, ESD, integrated care) and environmental factors (eg, physical, natural, and built) to identify and capture the most relevant and up-to-date research, policy, and gray literature. A systematic process will be used for item searching, selection, screening, coding, critical appraisal, and synthesis, building on methods outlined by Arksey and O’Malley [35] and recommendations by Levac et al [36].

#### Participants

Second, we will perform individual interviews with a strategic sample of 25 recruited stakeholders: people with stroke (n=5), significant others (n=5), health care professionals (n=10), and care managers (n=5). We will recruit people with stroke and significant others through stroke wards and outpatient wards. Persons with stroke meet the inclusion criteria if they have a diagnosed moderate stroke and have a focal neurological deficit when discharged, are 18 years or older, and can communicate and formulate answers to questions. We will also ask the persons with stroke to name a significant other if possible. We will recruit health care professionals (ie, occupational therapists, physiotherapists, nurses, and health care assistants) and care managers through collaboration with stroke units and municipalities that deliver home rehabilitation after stroke in Sweden. We will strive to balance the different occupational groups from the whole chain of care (ie, professionals employed in regions, as well as in municipalities).

#### Interviews

An interview guide will be designed by the research team, with the details aimed at the different target groups. First, participants will be encouraged to discuss their own rehabilitation experiences at home after a stroke, with a particular focus on the physical environment and participation. Second, participants will be invited to (1) discuss the results of the review; (2) comment on their views and understanding of critical environmental factors; and (3) recommend optimized strategies that include the environment and are likely to improve rehabilitation outcomes after stroke (eg, increased participation, self-efficacy, and mobility outside the home). For the persons with stroke, we will also use place-mapping. This is a participatory visualization technique that has been developed for studying persons with stroke. By reflecting on important places and modes of transport together, the interviewers and participants develop a place map together. Through the participatory process, the researchers can ensure that the collected data are comprehensive and valid. A place map represents the participant’s activity space in a schematic manner (see Meijering et al [14] for a detailed description of the method). The interviews will be audio recorded and transcribed verbatim.

#### Analysis

We will use qualitative content analysis [37], facilitated by the NVivo software (Lumivero) [38] to analyze the data. The output from the knowledge synthesis is a specification of important environmental factors for a person-centered rehabilitation process and implementation strategies.

### Addressing Research Question 2: Co-Designing the Intervention

#### Participants

In a second step, the same participants (who participated in the interviews) will be invited, with additional recruitment in case of dropout. The participants will then be divided into 2 groups.

#### Co-Designing Workshops

The 2 groups will participate in workshops in a co-designing process [39] guided by the “double diamond” method [40]. This means that we together with the participants (1) “discover,” that is, develop a deeper understanding of the problem or challenges that exist, (2) “define,” further define the problem and brainstorm about potential solutions, (3) “develop,” develop a prototype or model, and (4) “deliver,” deliver a model. The method has been frequently used in the research group. The 2 parallel co-design processes will encompass 3 half-day workshop meetings (in person or via the web), facilitated by people in the research team. We will use an iterative process of collecting data, moving between data collection, analysis, modifications to the intervention, and then further data collection. Data will be collected through participatory observations (ie, reactions, interactions, and potential conflicts), interviews, field notes, and photos of, for example, notice boards used during the workshop. The meetings will be audio recorded.

#### Analysis

Meeting minutes, interviews, and field notes will be transcribed and analyzed between workshop meetings using qualitative content analysis to reveal patterns or underlying themes in the content [37]. Findings will be brought to the following meeting. The output from co-designing the intervention is a first draft of the practice model and implementation plan.

### Addressing Research Question 3: Refining the Intervention

#### Participants

To refine the developed practice model and produce an intervention and implementation plan (output of the project), we will collect data from a series of focus groups with decision makers in health care and spatial planning. A notable advantage of this approach lies in the collaborative dynamics created within a group [41], fostering momentum and facilitating the concurrent emergence of opinions, beliefs, feelings, and attitudes alongside individual experiences [42]. We will recruit 8 people for each focus group (n=16) [42], through the research group’s established networks. Heterogeneity will be attained in diverse occupations, age, sex, and geographical factors (ie, urban or rural) to mirror both similarities and differences to enable diverse perspectives to emerge.

#### Focus Groups

A total of 2 focus groups with decision makers in health care and spatial planning, such as officials responsible for the operation of health care environments in the region and municipalities, will be formed and meet 2 times each. The interview guide will be developed based on the draft from the co-design phase. In total, 2 moderators will lead each group; 1 will lead the discussions; and 1 will observe group interactions and listen to the discussions. The observing moderator will take field notes on settings and group dynamics, as well as pose clarifying questions. At the end of each focus group session, the observing moderator will give a summary of the core issues discussed and allow participants to comment and suggest amendments. The meetings will be audio recorded. After round 2, audiotapes will be transcribed verbatim.

#### Analysis

Analyses will be accomplished according to Krueger and Casey [42]. The moderators will read the transcripts and listen to the recordings to get a feeling for the dynamics of the discussions and independently code the data. After the coding, the units of meaning will be categorized, and then themes will be formulated for further analysis.

### Ethical Considerations

All parts of InHome will be conducted following the Helsinki Declaration of Ethics in research. Ethical approval for the first study, the knowledge synthesis, was obtained from the Swedish Ethical Review authority in October 2023 (ID 2023-05164-01). Data will be registered and stored under the Personal Data Act and Dalarna University rules. We will comply with international agreements and rules. A data management plan will be developed according to Dalarna University rules. The individual data will be treated confidentially and if participants wish to withdraw their participation, their data will be removed from the database. Data from all phases will be treated on a general level, that is, individuals will not be identified in any publications. Finally, data of personal information and registration of databases will follow the General Data Protection Regulations. The research group has extensive experience in performing research on persons with frail health such as older persons or persons with neurological illnesses. Informed consent from the person considered to be included in the study will be obtained. Written and oral information on the study purpose and what participation would entail will be given before the informed consent. No compensation will be provided. The participants will also be informed about confidentiality and their right to withdraw from the study at any time.

## Results

This 4-year project commenced in January 2023 and will continue until December 2026. The scoping review is ongoing, and the results are, as of April 2024, currently being analyzed. The interviews, based on the results, started during the last quarter of 2023. The co-designing workshops, based on the interviews, are planned for autumn 2024, and refinement of the intervention in spring 2025. A final practice model and implementation plan is planned to be achieved in autumn 2026.

## Discussion

### Principal Findings

This project sets out to coproduce a new practice model that integrates environmental factors in rehabilitation after a stroke for a more person-centered service. We expect that the new model will lead to improved quality of life and increased reintegration in society for the large group of people who are rehabilitated at home after a stroke. In the long run, the knowledge from the project contributes to an increased awareness of the importance of the physical environment for sustainable health care and thus supports improved, strategic, and sustainable planning of living environments.

In Sweden and many other countries, care and rehabilitation are increasingly transferred to the local community and people’s ordinary homes [43]. The Government and Sweden’s Municipalities and County Councils (Sveriges Kommuner och Regioner) have emphasized the need to address the physical environment’s potential for the success of this new reform of health care. Even so, research seldom considers the role of the physical environment; instead, the focus is on how biomedical and a person’s physical functions influence participation and life after stroke [5]. Given that current rehabilitation models are far from adequately supporting persons with stroke after hospital discharge [44], future research and stroke health care policy initiatives should focus on the diverse and long-term needs of this population [45] and consider how the environment can be integrated to support these needs [5]. This is important because stroke survivors constitute a large group in society and today many of them report feeling abandoned and excluded from decision-making and rehabilitation planning, demonstrating a strong need for more person-centered approaches [5,15,16].

The qualitative data from the interviews will provide valuable insights into which strategies and environmental factors to focus on to improve rehabilitation outcomes after stroke (eg, increased participation, self-efficacy, and mobility outside the home). This participatory approach addresses the often-reported gaps in patient engagement in care and rehabilitation planning described above, fostering a model that is both practical and implementable. Moreover, the co-design process will occur in several iterative activities to inform the practice models’ development, design, and evaluation. It enables an accumulation of knowledge and increased mutual understanding between stakeholders and researchers and contributes to nonlinear learning that provides solid knowledge production [46]. Participation is crucial for designing user-friendly new methods, processes, or products as people participate in shaping models that represent their needs, experiences, and preferences.

Turning to place mapping, previous research has shown that this method is useful for gaining information about a person’s important places that are no longer accessible [14]. Our approach to using this method as part of the interviews will improve our understanding of barriers in the physical environment, as well as enhance the validity of the in-depth interview data. Moreover, using visual representations of a person’s activity space can facilitate a dialogue around meaningful places and how health care best can support access to them.

In Sweden, a high prevalence of environmental barriers in ordinary housing has been shown, and since persons with stroke often have functional limitations, they might experience accessibility problems, which can hinder activities in daily life and thus have negative consequences for health and well-being [47,48]. Many barriers in the home can easily be removed yet a large proportion of persons poststroke (range 5%-39%) report home adaptation needs as unmet [5]. Unmet needs related to access to transportation and mobility are also evident in the literature, for example, [49] highlighting a need for considering and supporting out-of-home mobility in the rehabilitation process.

The dissemination of results includes a range of activities such as conferences and seminars and published results in traditional open-access high-impact journals, and popular science articles in public press. In addition, our research method involves a continuous and integrated knowledge translation process because the project will be carried out in close collaboration with relevant actors. It enables knowledge and competence sharing between researchers and users of the results. We will also use our established networks of end user organizations for stroke care to spread findings in newsletters and on the organizations’ websites.

### Strengths and Limitations

InHome is conducted in Sweden, but the knowledge gained from the project is expected to have international health care policy relevance. Hence, the trend of shorter hospital stays, and continued care and rehabilitation is a global phenomenon that requires organizational change, as well as new approaches to meeting with persons with stroke and their relatives.

Our goal is to develop a practical and implementable model that is based on users’ needs. Co-designing is a fruitful method but there is a risk it is found too demanding. The participants may also come to insight into shortcomings in their rehabilitation process, but this is something we are prepared for. In addition, following MRC and PROCEDURES will support the quality and standardization of the development.

The small sample can be seen as a limitation. However, the focus of this project is on developing a new practice model; future studies with larger sample sizes are needed to evaluate the intervention through stages of feasibility testing and to establish effects and cost-effectiveness, knowledge needed for implementation (MRC). Finally, we will include participants with stroke receiving ESD, meaning they had mild to moderate stroke; experiences of those with severe stroke are yet to be investigated.

### Conclusions

InHome contributes with a prominent missing puzzle to optimize the rehabilitation process by adding a strong focus on user engagement combined with the integration of different aspects of the environment. The goal is to improve the quality of life and increase reintegration in society for the large group of people living with the aftermath of a stroke. InHome goes beyond traditional rehabilitation models that focus on physical functions and less on the whole life situation and opportunities to live an active life. We expect that the co-design method will make the model feasible and sustainable. In addition, the knowledge from the project will contribute to an increased awareness of the importance of the physical environment for sustainable health care. The findings will lay the foundation for future upscaling initiatives.

## Appendix

Multimedia Appendix 1 Assessment of application.

## Abbreviations

ESD: early supported discharge
ICF: International Classification of Functioning, Disability, and Health
MRC: Medical Research Council
PRODUCES: Problem Objective Design (end-) Users Co-creators Evaluation Scalability

## Data Availability

The data sets generated during this study are not publicly available due to privacy or ethical reasons, but anonymized data are available from the corresponding author on reasonable request.
