# Supplementary material for: Rehabilitation at Home With the Development of a Sustainable Model Placing the Person’s Needs and Environment at Heart: Protocol for a Multimethod Project
Source: JMIR Res Protoc. 2024 Jul 23;13:e56996. doi: 10.2196/56996 (PMC11303889; doi:10.2196/56996)
Supplement: Multimedia Appendix 1 [file resprot_v13i1e56996_app1.pdf]

FORMAS

Beredningsgruppens yttrande

**2022-00584**	**Maya Kylén**	**Beredningsgrupp: Brg2208**

**Utlysningsnamn:** Årliga öppna utlysningen 2022	**Bidragsform:** Forskare tidigt i karriären
**Projekttitel (svenska):** Rehabilitering i hemmet - utveckling av en hållbar modell som sätter personens behov och miljön i centrum (InHome)	**Sökt inriktning:** Forskarinitierad

Vetenskaplig frågeställning 6

1 - Insufficient, 2 - Poor, 3 - Acceptable, 4 - Good, 5 - Very Good, 6 - Excellent, 7 - Outstanding

Metod och genomförande 7

1 - Insufficient, 2 - Poor, 3 - Acceptable, 4 - Good, 5 - Very Good, 6 - Excellent, 7 - Outstanding

Vetenskaplig kompetens 7

1 - Insufficient, 2 - Poor, 3 - Acceptable, 4 - Good, 5 - Very Good, 6 - Excellent, 7 - Outstanding

Frågeställningens samhällsnytta och kommunikation av resultat 7

1 - Insufficient, 2 - Poor, 3 - Acceptable, 4 - Good, 5 - Very Good, 6 - Excellent, 7 - Outstanding

Slutbedömning (max 1 000 tecken inklusive mellanslag) 7

1 - Insufficient application, 2 - Poor application, 3 - Acceptable application, 4 - Good application, 5 - Very good application, 6 - Excellent application, 7 - Outstanding application

This is an outstanding application on an underexplored issue with great societal value. The proposal has a very high scientific relevance and is linked to ongoing state of the art. The theory and method are well-described, and the proposal includes a precise and realistic work plan. The scientific competence of the research team is strong and well put together to execute the project. Their track record is excellent, and they have highly influential and disseminated knowledge about the research topic. Both the societal importance of the study and communication plan are well documented. This makes it convincing that the results will have social impact. Overall, the project promises outstanding findings that will give novel and needed contribution to the development of a new model that integrates environmental factors in rehabilitation after a stroke.
